# Differential Release and Phagocytosis of Tegument Glycoconjugates in Neurocysticercosis: Implications for Immune Evasion Strategies

**DOI:** 10.1371/journal.pntd.0000218

**Published:** 2008-04-09

**Authors:** Jorge I. Alvarez, Jennifer Rivera, Judy M. Teale

**Affiliations:** 1 Department of Microbiology and Immunology, University of Texas Health Science Center at San Antonio, San Antonio, Texas, United States of America; 2 Department of Biology and South Texas Center for Emerging Infectious Diseases, University of Texas at San Antonio, San Antonio, Texas, United States of America; Queensland Institute of Medical Research, Australia

## Abstract

Neurocysticercosis (NCC) is an infection of the central nervous system (CNS) by the metacestode of the helminth *Taenia solium*. The severity of the symptoms is associated with the intensity of the immune response. First, there is a long asymptomatic period where host immunity seems incapable of resolving the infection, followed by a chronic hypersensitivity reaction. Since little is known about the initial response to this infection, a murine model using the cestode *Mesocestoides corti* (syn. *Mesocestoides vogae*) was employed to analyze morphological changes in the parasite early in the infection. It was found that *M. corti* material is released from the tegument making close contact with the nervous tissue. These results were confirmed by infecting murine CNS with ex vivo–labeled parasites. Because more than 95% of NCC patients exhibit humoral responses against carbohydrate-based antigens, and the tegument is known to be rich in glycoconjugates (GCs), the expression of these types of molecules was analyzed in human, porcine, and murine NCC specimens. To determine the GCs present in the tegument, fluorochrome-labeled hydrazides as well as fluorochrome-labeled lectins with specificity to different carbohydrates were used. All the lectins utilized labeled the tegument. GCs bound by isolectinB4 were shed in the first days of infection and not resynthesized by the parasite, whereas GCs bound by wheat germ agglutinin and concavalinA were continuously released throughout the infectious process. GCs bound by these three lectins were taken up by host cells. Peanut lectin-binding GCs, in contrast, remained on the parasite and were not detected in host cells. The parasitic origin of the lectin-binding GCs found in host cells was confirmed using antibodies against *T. solium and M. corti*. We propose that both the rapid and persistent release of tegumental GCs plays a key role in the well-known immunomodulatory effects of helminths, including immune evasion and life-long inflammatory sequelae seen in many NCC patients.

## Author Summary

Neurocysticercosis (NCC) is a disease caused by the larval form of a tapeworm parasite that preferentially migrates to the brain. It is characterized by a long asymptomatic period thought to result from the parasite's ability to evade host immunity. To date, the mechanisms of host–parasite interaction before symptoms develop remain unknown. In this study we evaluate by multiple immunofluorescent techniques distinct stages of the infection, making use of a murine model that closely resembles the disease process observed in humans. We discovered that the array of molecules secreted by the parasite varies according to the phase of infection studied. Early in infection, the parasite permanently sheds distinct molecules, allowing a rapid establishment in the brain. As the infection ensues, the continuous release of different molecules appears to facilitate the persistence of the parasite by downregulating molecules involved in its recognition and destruction. Loss of such molecules when the parasite dies after drug treatment may explain sudden inflammatory responses in patients. Characterization of these molecules will lead to advances in our understanding of the complex immunoregulatory mechanisms used by parasites and to new approaches for therapeutic strategies.

## Introduction

Neurocysticercosis (NCC), caused by the larval form of the tapeworm *T. solium*, is one of the most common parasitic infections of the CNS worldwide [1],[2]. Although the metacestodes reach their mature size within a few weeks, evidence indicates that prior to clinical manifestations, there is a long asymptomatic period (months or even years) thought to be the result of numerous mechanisms that the organism uses to modulate and inhibit the immune response [3]. Eventually, clinical symptoms appear and include headache, seizures, and hydrocephalus that can be devastating and lifelong. Symptoms are normally associated with chronic inflammatory responses suggesting a source of persistent antigen [2],[4]. Thus, immune evasion and persistent antigen appear to be important characteristics of this disease process.

Our laboratory has developed a mouse model of NCC using the similar cestode *M. corti*. This organism does not infect the brain naturally as does *T. solium* and is therefore missing the normal progression of the immature larva to the more mature cysticerci and potential antigenic changes. Another drawback is that the parasite is able to proliferate and invade brain tissue. Nevertheless, our multiple studies of CNS infection-induced immune responses using *M. corti* have resulted in many findings that parallel that of the natural infection in humans and pigs [5]–[11]. Therefore, it remains an important model for helping to dissect mechanisms of disease pathogenesis.

In the last two decades, major interest has been placed in understanding both the molecular nature of the antigens associated with disease and elucidation of their role in immune response and vaccine development [12]–[18]. It has been shown that the glycosidic portion of glycoproteins and other glycoconjugates (GCs) expressed by *T. solium* metacestodes are highly antigenic, being recognized by serum from infected patients and mainly studied as potential targets in serological diagnosis [14],[16],[19]. These GCs may also play an important role in parasite-host interactions as well as in the modulation of the immune response [3]. Part of this strategy concerns the tegument or external surface molecules present on the parasite. The tegument of helminths such as *T. solium* and *M. corti* consist of a syncytium organized into two zones; an anucleate area called distal cytoplasm and a nucleated area known as the proximal cytoplasm [20]–[22]. The distal cytoplasm contains some mitochondria, vesicles and discoidal bodies that appear to be involved in the formation and replacement of the outer-surface membranes [22],[23]. In helminths, the external surface is dynamically responsive to changing host environments or immune attack and under these adverse circumstances can rapidly shed layers [24]. Therefore, surface bound antibodies, complement and activated immune effector cells can be sloughed off. Material that is released from the tegument can act as a smokescreen diverting the immune response to static deposits of antigen separated from the parasite itself [24]. In addition, the high antigenicity of *T. solium* GCs may play a role in hypersensitivity reactions [11] and ultimately to pathological symptoms and disease.

To better understand the role of tegument GCs, it is important to determine their localization and potential dissemination during the infectious process. As NCC is an infection characterized by a long asymptomatic period, the analyses of the early events in the infection process are difficult to perform. Therefore a mouse model that closely resembles the infection in humans is particularly useful for studying the fate of parasitic antigens early in infection as well as in the later phases of this process. These results were validated by the study of tegument GCs in specimens from porcine and human NCC.

## Materials and Methods

### Animals

Female BALB/c mice 3–5 wk old were purchased from the National Cancer Institute Animal Program (Bethesda, MD). Animal experiments were conducted under the guidelines of the University of Texas System, The U.S. Department of Agriculture, and the National Institutes of Health.

### Parasites and inoculations

*M. corti* metacestodes were maintained by serial intraperitoneal inoculation of 8 to 12 wk old female BALB/c mice. Intracranial inoculations were performed as described previously [7]. Briefly, a 25 gauge needle was inserted 2 mm deep into the bregma region where there is space between the skull and the brain to ensure no penetration of the nervous tissue. Mock control mice were injected with 50 µl of sterile Hank's Balanced Salt Solution (HBSS). Before intracranial inoculation, mice were anesthetized intramuscularly with 100 µl of anesthetic cocktail containing 100 mg/ml ketamine and 20 mg/ml rompum (Laboratory Animal Resource, University of Texas Health Science Center (UTHSC), San Antonio TX). Before sacrifice, animals were anesthetized with 100 µl of cocktail and perfused through the left ventricle with 15 ml cold phosphate buffered saline (PBS) pH 7.4. Animals were sacrificed after 1d, 3d, 1 wk, 3 wks and 5 wks after inoculation.

### Human and porcine tissue source, processing and histological analysis

Metacestodes extracted from naturally infected pigs and brain, skeletal, or cardiac muscle infected with parasites were collected, fixed in neutral buffered formalin (10% v/v formaldehyde, 29 mM NaH2PO4, 45 mM Na2HPO4) for 48 h and embedded in paraffin. Brain specimens from four symptomatic NCC cases characterized by the presence of inflammatory infiltrates surrounding the parasite were obtained from Hospital Universitario San Jose in Popayán, Colombia. Medical information explaining why these patients underwent surgery was limited and no data was available regarding corticosteroid treatment before surgical intervention. Research using these patient specimens complied with all relevant federal guidelines and institutional policies. Human samples were processed similar to those from pigs. Serial 5-µm-thick sections were mounted on silane preparation slides (Sigma, St. Louis, MO) and used for histological and immunofluorescent microscopy procedures. The tissue sections were stained with hematoxylin and eosin (H&E) to determine the stage of viability of the parasite and the extent and type of leukocyte infiltration. H&E slides were analyzed with a Leica DMR microscope (Leica Microsystems, Wetzlar Germany). Images were acquired using a cooled CCD SPOT RT camera (Diagnostic instruments Inc, Sterling Heights, MI). The images were processed and analyzed using Adobe Photoshop 7.0 (Adobe, Mountain View, CA). The specimens from pigs were classified according to the stage of infection as previously described [6],[25]. Brains removed from mice intracranially infected with *M. corti* were perfused, embedded in O.C.T. resin (Sakura, Torrance, CA) and snap frozen. Serial horizontal 10 µm cryosections of the whole brain were placed on xylene prep slides (Sigma-Aldrich, St. Louis, MO). One in every five slides was stained with H&E to determine the location of cellular infiltrates around parasites and distant from them. The remainder of the slides was air dried overnight and fixed in fresh acetone for 20 s at rt. Acetone-fixed sections were wrapped in aluminum foil and stored at −80°C or processed immediately for immunostaining.

### Transmission electron microscopy

Parasites obtained from the peritoneal cavity and brains from mice (*n* = 3) infected for 1 day and 3 days were processed for analysis by transmission electron microscopy (TEM). After being removed from the peritoneal cavity, parasites were cultured in saline solution for 24 hrs and fixed in a phosphate-buffered mixture (84 mM NaH2PO4, 68 mM NaOH) of 4% formaldehyde (v/v) and 1% glutaraldehyde (v/v). The brain was perfused with 15 ml of cold PBS and then fixed in the same solution. Parasites and brain samples of approximately 1 mm^2^ were cut in ultrathin 90-nm sections that were collected on a 150-mesh copper grid and stained with saturated aqueous uranyl acetate and Reynolds lead citrate (Electron Microscopy Sciences, Fort Washington, PA) for microscopic analysis. Photographs were taken using a JEOL 1230 electron microscope (JEOL, Peabody, MA) with an accelerated voltage of 80 kV.

### Labeling of parasites with fluorochrome conjugated hydrazide

GCs present in the tegument of *M. corti* were labeled by oxidation of cell-surface glycoproteins and polysaccharides followed by reaction with a membrane-impermeant hydrazide (Molecular probes Handbook – Eugene, OR). Labeling of hydrazide conjugated with Alexa 488 was done using two techniques. The first was the periodate method in which parasites extracted from animals previously infected through the intraperitoneal (IP) route were washed in PBS-0.05% Tween 20 and resuspended in a solution of 0.1 M sodium acetate, 1 mM sodium periodate pH 5.5. Then, the parasites were incubated 20 min at 4°C with gentle rotation, and the reaction was stopped by adding 0.1 mM glycerol. After washing with PBS-0.05% Tween 20 they were resuspended in 1 mM Alexa 488 hydrazide and incubated 2 hrs at room temperature. Parasites were washed in PBS-0.05% Tween 20 and mounted in OCT for microscopic analysis. The second method used the INFLUX kit purchased from Molecular Probes (Eugene, OR). Parasites were extracted as previously described and after washing in PBS-0.05% Tween 20 they were labeled following the manufacturer's protocol. After labeling they were embedded in OCT and processed for microscopy. In addition, parasites (approximately 70) labeled using both techniques were intracranially (IC) injected into 3–5 wk old mice (*n* = 3) as described above. Animals were sacrificed 12 hrs, 24 hrs and 5 days PI, and samples were processed as previously described. Fluorescence was visualized in a Leica DMR epifluorescent microscope (Leica Microsystems, Wetzlar Germany). Images were acquired using a cooled CCD SPOT RT camera (Diagnostic instruments Inc, Sterling Heights, MI), and they were processed and analyzed using Adobe Photoshop 7.0 (Adobe, Mountain View, CA).

### Generation of anti-gp12 antiserum

*T. solium* metacestodes were processed to obtain a preparation of glycoproteins as previously described [16],[26]. Briefly, the homogenate of the metacestodes was run through a *L. culinaris* affinity column and enrichment of the 12-kDa glycoprotein (gp12) was done by electroelution. Purification of gp12 for immunization was done using a 12% SDS-PAGE Prep-Cell system (BioRad). Fractions containing the 12-kDa antigen were pooled, concentrated by ultracentrifugation, and quantified by the Bradford method (BioRad) [27]. The gp12 was detected using silver-stained gels and western blots utilizing sera from patients with NCC. Then, 200 mg of gp12 emulsified with a suspension of RIBI adjuvant (Sigma, St. Louis, Missouri) was used to immunize a New Zealand rabbit. The inoculations were done at intradermal, subcutaneous, and intramuscular sites. A similar procedure was used for 2 booster injections on days 14 and 28. The serum used in this study was obtained at day 42.

### Antibodies and lectins

The lectins Isolectin-B4 (IB4) conjugated with Alexa 546, wheat germ agglutinin (WGA) conjugated with Alexa 350, concavalin A (ConA) conjugated with Alexa 488 and *Arachis hypogaea* lectin (PNA) conjugated with Alexa 488 were purchased from Molecular Probes (Eugene, OR). Monoclonal antibodies against *M. corti* were raised against supernatant collected after in vitro incubation of *M. corti* metacestodes for 3 days. Two anti-*M. corti* supernatant (MCS) monoclonal antibodies were used, MCSγ3 and MCSγ1. Previous studies have determined that the supernatant contains several molecules that are being actively secreted by the parasite [28]. The antibodies were labeled with different fluorochromes using the kit from Molecular Probes (Eugene, OR). The rat anti-CD11b antibody conjugated with phycoerithrin (PE) was purchased from BD pharmingen (San Diego, CA). Primary anti-human antibodies directed against CD3 for T cells, CD8 for cytotoxic T cells, CD20 for B cells, CD68 for macrophages/microglia, tryptase for mast cells and MHC-II were purchased from DAKO (Carpinteria, CA). The secondary antibodies used in paraffin sections were biotinylated and cross-absorbed with human serum proteins. These included an affinity-purified goat anti-mouse IgG and a goat anti-rabbit IgG purchased from KPL (Gaithersburg, MD). The optimal conditions for each antibody were established in human tonsils obtained from patients with chronic tonsillitis or with normal human brain specimens.

### Immunofluorescence microscopy

Labeling with fluorochrome conjugated lectins was used to determine the sugar composition in the tegument of *M. corti* and *T. solium*. Human and porcine samples were deparaffinized in multiple xylene washes and rehydrated in decreasing solutions of ethanol. Frozen mouse sections were fixed in −20°C acetone for 10 min, −20°C ethanol 70% for 5 min and then hydrated in PBS. Paraffin embedded and frozen samples were processed similarly. Fluorochrome labeled lectins with specificity to different carbohydrate moieties were used in an immunofluorescent assay in multiple combinations. IB4 binds specifically to acetyl-D-galactosamine ends and α-D-galactosyl residues, and WGA binds to N-acetylglucosaminyl residues. Also used in these studies were ConA which binds to α-mannopyranosyl and α-glucopyranosyl residues and PNA, which binds to terminal β-galactose residues. The specific binding of the lectins in these studies was confirmed by using excess amounts of the relevant sugars during staining with fluorochrome-labeled lectins which resulted in the absence of staining.

Immunofluorescence staining in paraffin sections using antibodies was performed as followed. Sections were deparaffinized and rehydrated as previously described. Unmasking of the target molecules was performed by incubation in antigen retrieval solution at 92°C for 30 to 60 mins, depending on the marker analyzed. Blocking of Fc receptors was done with 10% serum from the species in which the fluorochrome conjugated antibody was generated. Sections were incubated with the primary antibody diluted in 3% species specific serum for one hr at rt. Sections were washed 7× for 3 mins each after incubation with the specified antibodies. Secondary antibodies were incubated for 30 mins at rt when necessary. Subsequently, the second set of primary antibodies with their respective secondary antibody or fluorochrome labeled lectins were incubated. Some of the reactions were enhanced with tyramide treatment (NEN Life Science Products, Boston, MA) following manufacturer's protocol. Sections were mounted using fluorsave reagent (Calbiochem, La Jolla, CA) containing 0.3 µM 4′,6′-diamidino-2-phenylindole dilactate-DAPI (Molecular Probes, Eugene, OR) if required. Fluorescence was visualized and analyzed as previously described. Immunofluorescence staining in frozen sections was done following the same protocol, but the antigen retrieval step was omitted. Immunofluorescence and immunohistochemistry in mouse frozen sections and human samples was performed as previously described [7],[11].

## Results

### Changes in the tegument of *M. corti* upon CNS infection

**Figure 1:**
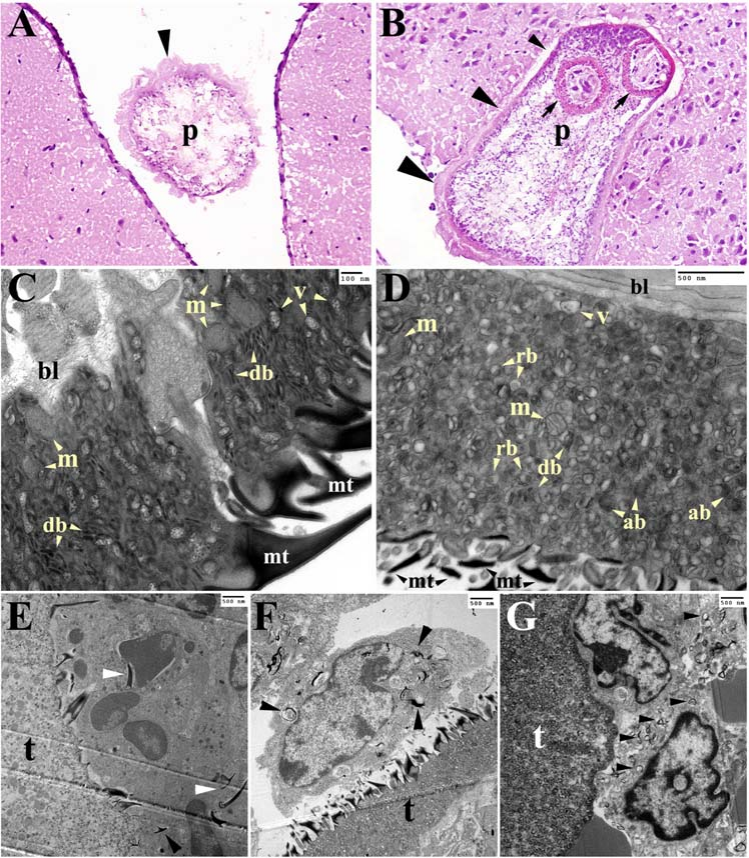
Changes in the tegument of *M. corti* metacestode during CNS infection. TEM images are showing the tegument distal cytoplasm (A) H&E staining of 1 day post-infection (DPI) brain showing a parasite (p) in the subdural space. The parasite's tegument (arrowhead) appears intact. 200× (B) H&E staining of *M. corti* penetrating the nervous tissue. Near complete loss of the tegument (small arrowhead), intermediate loss (medium arrowhead) and no loss (large arrowhead) can be observed during the invasion process. Arrows depict the scolex tegument. 2 DPI - 200× (C) TEM of *M. corti* tegument extracted from the peritoneal cavity showing vesicles (v) and high number of mitochondria (m) and discoidal bodies (db). Microtriches (mt) appear intact (D) TEM of *M. corti* tegument after 1 day of CNS infection showing residual bodies (rb) and autophagic-like bodies (ab). The number of vesicles (v), mitochondria (m) and discoidal bodies appears lower. Microtriches (mt) are smaller or broken compared to parasites extracted from the peritoneal cavity (E) Microtriches (arrowheads) phagocytosed by immune cell interact with parasite tegument (t) 1 DPI. The vesicular appearance of the tegument previously described in D is also seen in this specimen (F) Apparent macrophage in vicinity of parasite's tegument (t) showing parasite structures in its cytoplasm (arrowheads) 3 DPI (G) Parasite tegument (t) surrounded by immune cells phagocytosing parasite derived material (arrowheads), 3 DPI. Note the lack of microtriches in the tegument.

The first focus of the study was to evaluate the early events of parasite invasion by using routine histological staining as hematoxylin and eosin (H&E) and transmission electron microscopy (TEM). One day after infection most of the metacestodes are located in the subdural (Figure 1A) and subarachnoid space, although some organisms can be found in the ventricular spaces. After 2 and 3 days metacestodes are found to actively invade the nervous tissue and exhibit a progressive loss of their tegument in areas in close contact with the nervous tissue (Figure 1B small arrowhead). In contrast, areas of the parasite still located outside of the tissue are thicker and appear to remain intact (Figure 1B large arrowhead). After 1 wk many of the metacestodes had completely invaded the tissue exhibiting a much thinner tegument on their surface (data not shown). TEM was used to obtain closer detail of the structural changes occurring in the metacestode at earlier stages of the infection. In order to detect changes in the tegument upon tissue invasion, metacestodes obtained from the peritoneal cavity of infected mice were incubated in saline solution for 24 hours and compared with those injected in the CNS. The distal cytoplasm from metacestodes obtained from the peritoneal cavity was characterized by the presence of vesicles, high numbers of discoidal bodies and large mitochondria (Figure 1C). In contrast, the distal cytoplasm of parasites lodged in the CNS for 1 d showed a high number of residual bodies and autophagic-like bodies (Figure 1D). The number of mitochondria, vesicles and discoidal bodies were reduced (Figure 1D), some of the microtriches were shorter or broken down (Figure 1D) and appeared in the cytoplasm of host cells surrounding the microorganism (Figure 1E). After 3 DPI the distal cytoplasm showed similar signs of stress, with increased vacuolization of the tegument (Figure 1F and 1G), and more microtriches detected in cells surrounding the parasite (Figure 1F). In some parasites, the majority of the microtriches were absent in the tegument, and the cells in the vicinity showed substantial phagocytosed material (Figure 1G).

### Different labeling techniques distinguish distinct molecules in the tegument

**Figure 2:**
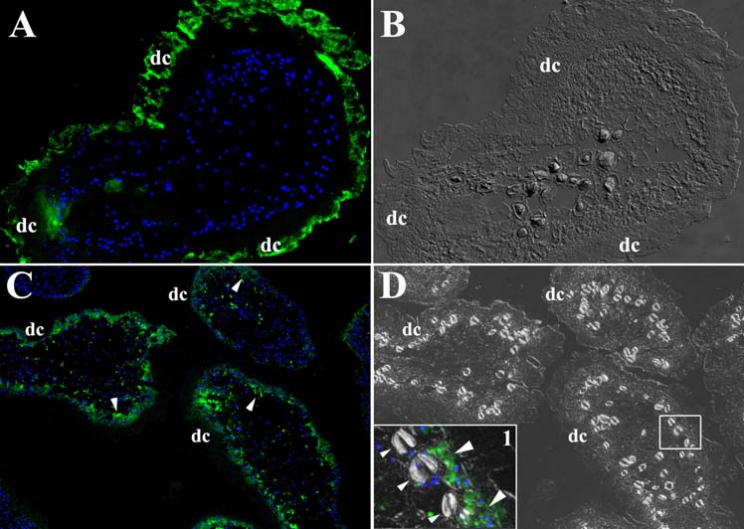
Peritoneal *M. corti* labeled with hydrazide Alexa 488. Nuclear stain is blue. (A) After periodate labeling, *M. corti* distal cytoplasm (dc) is highly fluorescent. Other areas of the parasite showed low to no fluorescent signal. 400× (B) DIC image of A confirming that hydrazide-Alexa 488 is coupled to molecules in the dc. 400× (C) Parasites labeled with the INFLUX kit show fluorescent signal in the proximal cytoplasm (arrowheads) but not in the dc. 200× (D) DIC image of C confirming that molecules labeled with the INFLUX kit are in the proximal cytoplasm and not in the dc. Inset 1 is showing that hydrazide-Alexa 488 labeled molecules are present in the proximal cytoplasm (large arrowheads), but not in the calcareous corpuscles (small arrowheads) or dc. Inset 1 is a DIC and fluorescence image 2.5 times magnified.

The tegument in helminths and nematodes is rich in carbohydrates and lipids [29],[30]. Therefore, using two methods the organism was labeled with hydrazide groups that are known to bind oxidizable aldehyde groups which are highly represented in molecules containing carbohydrates and lipids [31]. *M. corti* metacestodes labeled using the periodate method showed strong staining in the tegument's distal cytoplasm (Figure 2A–2B) but not in other areas. Interestingly, the INFLUX kit mainly labeled the proximal cytoplasm (Figure 2C–2D), but not the calcareous corpuscles (Figure 2D inset) or the distal cytoplasm labeled with the periodate technique.

### *M. corti* releases both distal cytoplasm and proximal cytoplasm molecules during infection

**Figure 3:**
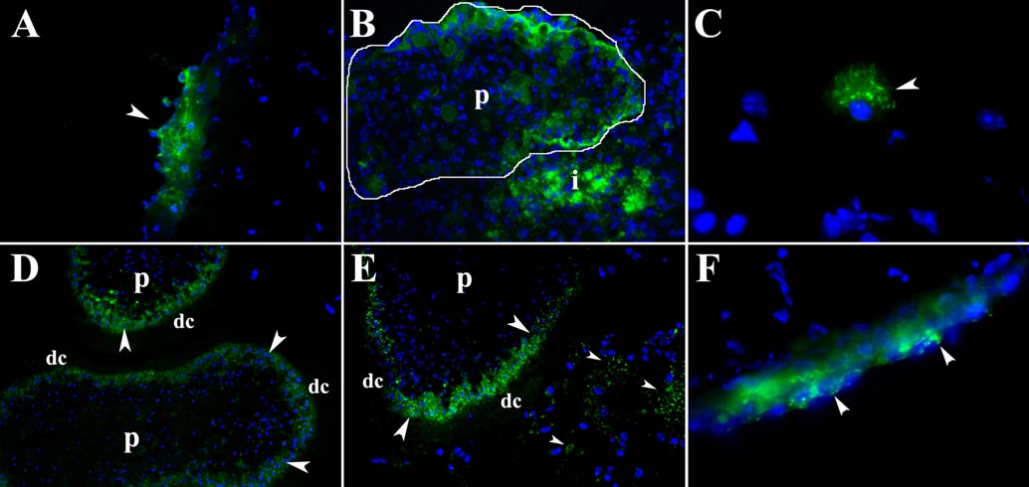
Hydrazide Alexa 488 labeled *M. corti* metacestode/GCs in mouse CNS. Pictures A to C are from parasites labeled with the periodate method, D to F were labeled with the INFLUX kit. Nuclear stain is blue. (A) Infiltrate in external leptomeninges showing accumulation of parasitic material (arrowhead) released from tegument 1 DPI. 400× (B) Non-viable parasite (p, circled) and associated infiltrate (i) containing hydrazide 488 associated molecules at 5 DPI. 400× (C) Phagocytic cell located in internal leptomeninges showing fluorescent staining in the cytoplasm (5 DPI 1000×) (D) Parasite (p) in internal leptomeninges showing signal in proximal cytoplasm (arrowheads), but not in distal cytoplasm (dc) 1 DPI. 400× (E) Parasite (p) located under external leptomeninges showing moderate fluorochrome signal in the proximal cytoplasm (large arrowheads). Some released material (small arrowheads) can be detected in the surroundings, but there is not a clear association with host cells (1 DPI 400×) (F) Infiltrate in external leptomeninges showing accumulation of parasitic material (arrowheads) released from proximal cytoplasm 5 DPI. 400×

To track distal cytoplasm vs proximal cytoplasm molecules during infection; female BALB/C mice were intracranially infected with *M. corti* metacestodes labeled by both methods and sacrificed at 12, 24 h and 5 d. After 12 or 24 h of infection the tegument material labeled with the periodate technique was mainly found in infiltrates located in external and internal leptomeninges (Figure 3A). After 5 days of infection the fluorescent labeling in distal cytoplasm was almost undetectable in most of the viable parasites. There were also metacestodes with strong fluorescent signal, but they were nonviable and contained host cells (Figure 3B). Distal cytoplasm material was mainly detected in cells with a macrophage-like morphology located in infiltrates of the external leptomeninges (Figure 3C).

*M. corti* metacestodes labeled with the INFLUX kit were also located in leptomeninges after 12 and 24 hours of infection. These parasites had a similar pattern of hydrazide-Alexa 488 distribution than before injection (Figure 3D), and some of them appeared to release proximal cytoplasm material in the periphery, although at this time most of this material did not seem to be phagocytosed by host cells (Figure 3E). After 5 days of infection the fluorescent signal coming from the proximal cytoplasm was partially lost, and it was detected in a few infiltrating cells located in external leptomeninges (Figure 3F). These results suggest that upon infection *M. corti* metacestodes released material predominantly from its distal cytoplasm and to a lesser extent from the proximal cytoplasm. In addition, the parasite material labeled by both techniques was released during infection.

### Lectin binding properties of *M. corti* prior to CNS infection

**Figure 4:**
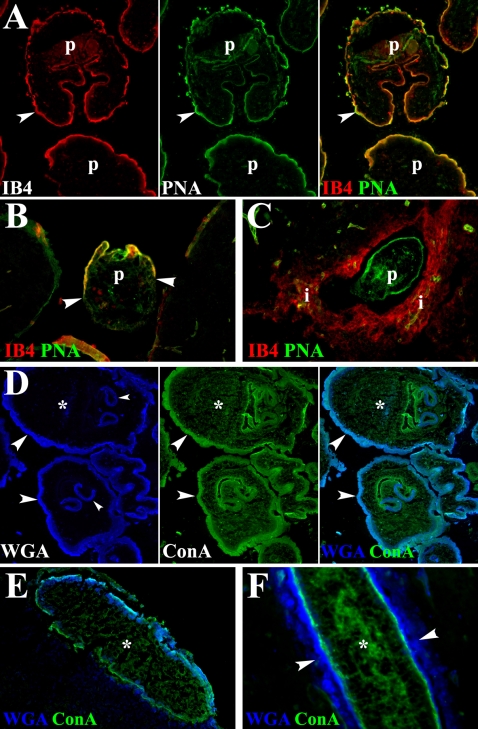
Release of tegument material in mice intracranially infected with *M. corti*. (A) Peritoneal *M. corti* (p) stained in the tegument (arrowheads) with IB4 and PNA. 200× (B) Parasite (p) invading nervous tissue 2 DPI. Tegument in contact with host tissue (arrowheads) has lost IB4 and PNA GC ligands. 200× (C) After 3 weeks of IC infection the parasite (p) is IB4 negative but the infiltrating cells are IB4 positive. PNA bound GCs are found in the whole parasite. 200× (D) *M. corti* tegument (large arrowheads) stained with WGA and ConA. Metacestode parenchyma (asterisks) is mostly stained with ConA. Scolex tegument (small arrowheads) is also stained with both lectins. 200× (E) *M. corti* metacestode in leptomeninges after 3 DPI. ConA binding material is present in tegument and parenchyma (asterisk), WGA binding material is released from the tegument (arrowheads). 200× (F) At 3 weeks of IC infection *M. corti* remains positive for ConA and WGA in the tegument, but a portion of the WGA binding material (arrowheads) continues to disassociate from the parasite 400×.

To further characterize the molecules released by *M. corti* upon infection, the lectin binding properties of the parasite were analyzed. *M. corti* metacestodes that are propagated intraperitoneally (IP) in BALB/C female mice were used. Acetyl-D-galactosamine ends and α-D-galactosyl residues, carbohydrates bound by IB4, were mainly found in the tegument's distal cytoplasm (Figure 4A). N-acetylglucosaminyl residues bound by WGA also stained predominantly the distal cytoplasm (Figure 4D), whereas terminal β-galactose residues recognized by PNA and α-mannopyranosyl and α-glucopyranosyl residues bound by ConA were present in both the tegument and in the parasite's parenchyma (Figure 4A and 4D respectively).

### Fate of lectin specific GCs after CNS infection

Mice were intracranially infected, sacrificed after different times PI, and brain sections analyzed for lectin specific binding. By 1 DPI, IB4 which primarily stains distal cytoplasm is progressively lost, particularly in areas of close contact with the nervous tissue (Figure 4B). PNA bound material was lost, but to a lesser extent (Figure 4B). Importantly, IB4 bound material was essentially absent from the parasite by 3 DPI and remained so throughout the infection process. In parallel, large numbers of cells surrounding the parasites can be seen positive for IB4 binding material (Figure 4C) by 1 and 3 wks PI. In contrast, PNA and ConA bound material were still present on the parasite (tegument and body) (Figure 4C, 4E and 4F) even 5 wks PI. Some blood vessels can be seen positive for PNA (Figure 4C) but this is not related to parasite released GCs as vessels are equally stained with the lectin in non-infected brain and mock infected controls. In contrast to IB4 bound material which is lost by 3 DPI, WGA bound material is constantly released by the parasite throughout the infection (Figures 4E (3 DPI) and 4F (3 WPI)), and it can be detected in infiltrates located far away from parasites (see below).

### GCs released by *M. corti* are phagocytosed by host cells

**Figure 5:**
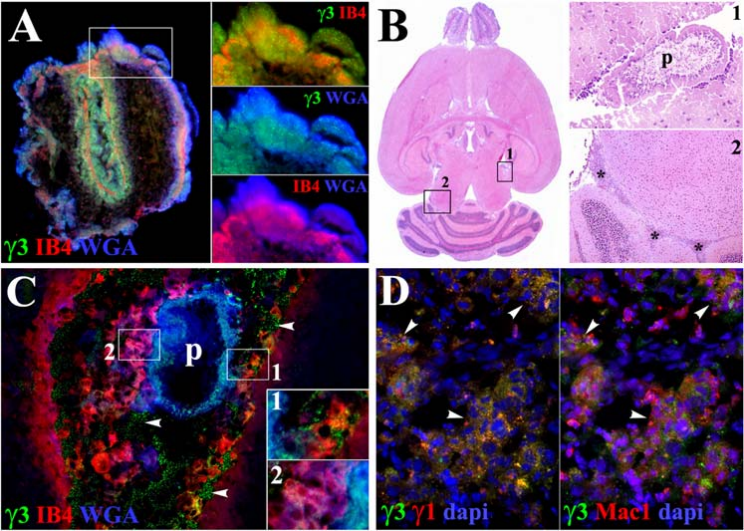
Host cells phagocytose *M. corti* GCs during the course of murine NCC. (A) γ3, IB4 and WGA staining colocalize in tegument and parenchyma of IP maintained parasite after multiple washes with HBSS (400×). Distinct combinations of lectin and γ3 staining in the tegument are shown in the right panels (B) H&E staining of mouse brain 3 wks PI (10×). Inset 1 (200×) is showing parasite (p) located in internal leptomeninges similar to that in C. Inset 2 (50×) shows perivascular infiltrates (asterisks) located distal to parasites, details of such infiltrates are shown in D (50×) (C) *M. corti* (asterisk) stained with WGA and γ3, but not IB4 in internal leptomeninges 3 wks PI. Infiltrating cells are double positive for IB4 and MCSγ3 (inset 1) and for WGA and IB4 (inset 2). Free parasite material is also detected (arrowheads). 200×. Inset 1 and 2 are 2.5 times magnified. (D) Inflammatory infiltrates (arrowheads) in internal leptomeninges showing γ3 and γ1 staining. Panel on the right shows that parasite material labeled by γ3 and γ1 is present in Mac1^+^ cells 3 wks PI. 400×.

An important finding of these studies was the apparent uptake of parasite GCs by infiltrating mononuclear cells. To confirm that GCs were parasite derived and not the result of upregulation of glycosylated molecules of activated leukocytes, monoclonal antibodies were generated against *M. corti* molecules. Two anti-MCS monoclonal antibody were used, MCSγ3 and MCSγ1. Both MCS antibodies labeled *M. corti* metacestodes in overlapping areas where IB4 and WGA bound. A representative image using MCSγ3 is shown in Figure 5A. After validation of the MCS antibodies, the release of parasite material was followed kinetically in animals with IC infection. It was found that after 1 wk of infection the MCS antibodies predominantly stained parasite material in the Virchow-Robin spaces and intraventricular areas (data not shown). In contrast, low levels of MCS^+^ parasite material were detected in infiltrating cells or parenchymal tissue. By 3 wks of infection larger numbers of cells containing MCS specific material were seen in infiltrating cells surrounding parasites (Fig 5B–5C). Interestingly, infiltrates located under meninges and far away from parasites had a great number of MCS positive cells (Figure 5B–5D). To determine the cell types involve in phagocytosis, different cellular markers were used. The majority of phagocytes appear to be macrophages (Figure 5D) indicated by staining with CD11b, although it is possible that some of these cells are microglia. In summary, these experiments confirm that the GCs detected by lectin binding are released by *M. corti* and this material is phagocytosed by host cells including areas in which parasites were absent.

### GCs in the tegument of *T. solium* metacestodes are also released during infection

**Figure 6:**
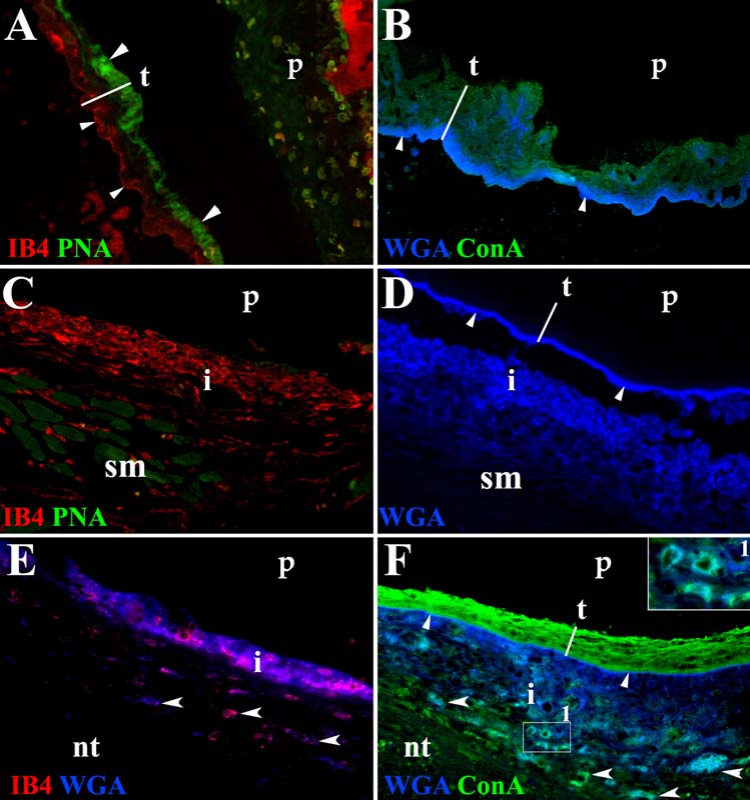
In situ staining of *T. solium* GCs present in the course of porcine and human NCC. Fluorochrome labeled lectins were used to detect distinct glycosidic conformations in the tegument of metacestodes infecting porcine and human tissue. (A) Stage I parasite (p) extracted from porcine skeletal muscle. In the tegument (t), IB4 labeled the distal cytoplasm (small arrowheads) and PNA labeled the proximal cytoplasm (large arrowheads). 200× (B) Same stage of p shown in A. WGA labeling predominated in distal cytoplasm (arrowheads) and ConA labeled the whole t. 200× (C) Stage II p in porcine skeletal muscle (sm). IB4, but not PNA stained infiltrating cells (i) located around the p. 400× (D) WGA stained distal cytoplasm (arrowheads) in stage III p lodged in skeletal muscle. WGA bound material is released from t and it is detected in the infiltrate (i) 400× (E) p in human nervous tissue (nt). IB4 and WGA bound material colocalize in the infiltrate (i) that surrounds the p and in some nervous cells (arrowheads, fucsia). 400× (F) ConA labeled the whole t and WGA the distal cytoplasm (small arrowheads) of p lodged in human nervous tissue (nt). Both lectins are released and colocalize with cells in the infiltrate (i, inset 1) and some nervous cells located in the vicinity of the metacestodes (large arrowheads, lighter blue) 400×

In order to validate the data obtained with *M. corti*, skeletal and cardiac muscle samples representative of different stages of infection in pigs [6],[25] were used to determine the GCs present in the tegument of *T. solium* and their changes during host-parasite interaction. Human brain specimens obtained from NCC patients after surgery were also utilized. *T. solium* metacestodes extracted from pigs and in stage I of the infection were frozen and subsequently used to determine the lectin binding profile in tegument. In stage I the parasite appears to be viable and it is accompanied by collagen deposition and few infiltrating cells [6]. IB4 staining in *T. solium* metacestodes indicates that the distal cytoplasm contains acetyl-D-galactosamine ends and α-D-galactosyl residues (Figure 6A). A similar pattern of staining was observed with WGA indicating the presence of N-acetylglucosaminyl residues (Figure 6B). In contrast, α-mannopyranosyl and α-glucopyranosyl residues, determined by ConA binding were observed in the whole tegument (Figure 6B). Finally, terminal β-galactose residues detected with PNA were only found in the proximal cytoplasm (Figure 6A).

In porcine and human NCC the material bound by WGA, IB4 and ConA, but not PNA is released by the parasite, and it seems to be taken up by the cells around the metacestode (Figure 6C to 6F) resembling the pattern of release seen in murine NCC (Figure 4). The released IB4 bound material is mainly found in the infiltrates surrounding the parasites (Figure 6C and 6E). In contrast N-acetylglucosaminyl residues bound by WGA are present in the infiltrates and tegument of parasites lodged in porcine skeletal muscle (Figure 6D) and human brain (Figure 6E and 6F). ConA bound material is detected in the whole tegument, particularly in the proximal cytoplasm and colocalizes with WGA in infiltrates and CNS cells in the vicinity of the parasite (Figure 6F).

### *T. solium* glycoproteins are taken up by phagocytes

**Figure 7:**
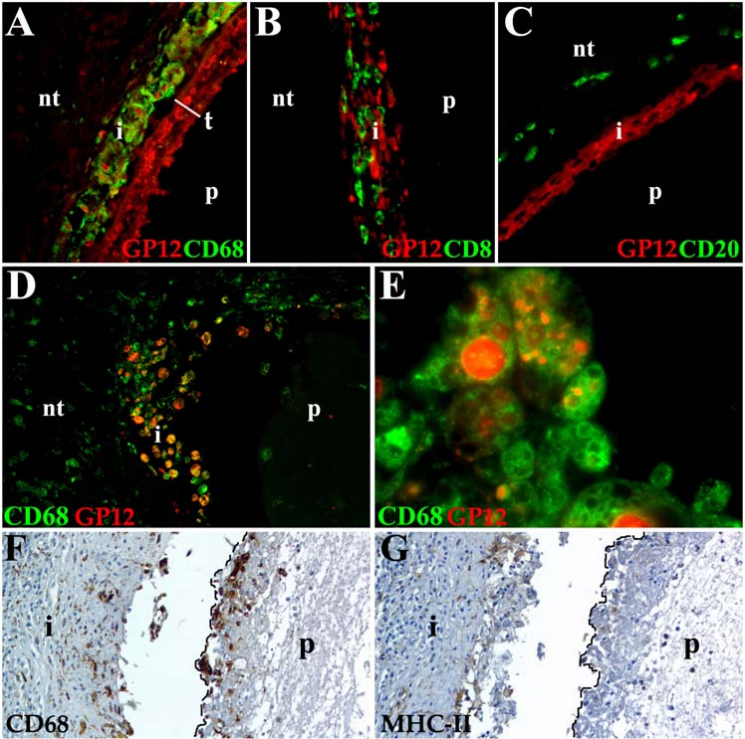
Host cells taking up *T. solium* GP12. Different leukocyte populations were detected with monoclonal antibodies and streptavidin-Alexa 488 (green), streptavidin-HRP (brown). Anti-GP12 polyclonal antibody was labeled with Alexa 546 (red). Samples A–C are from non-granulomatous lesions and D–G from samples with granuloma formation. Nervous tissue (nt). (A) Anti-GP12 strongly labels the whole parasite (p) tegument (t) and the macrophages (CD68) present in the infiltrate (i) 400×. (B) CD8 T cells do not colocalize with anti-GP12 in the i. The p represents the space where the parasite was lodged. 400× (C) Anti-GP12 stains some cells in the i, but no B cells (CD20). 400× (D) p surrounded by i showing macrophages co-localizing with anti-GP12 (orange-yellow color). 400× (E) Epithelioid histiocytes and giant cells showing anti-GP12 staining in most of the vacuoles present in their cytoplasm. 1000× (F) Macrophages and multinucleated giant cells (dotted line) surrounded the p and located in i forming granuloma. 400× (G) Low to undetectable expression of MHC-II in macrophages (dotted line) adjacent to the p. Moderate MHC-II expression is detected in the i. 400×.

To confirm that GCs detected by lectin staining are released from *T. solium* tegument and then ingested by immune cells, a polyclonal antibody against a 12 kD glycoprotein (GP12) from *T. solium* was used [27]. This antibody was chosen because GP12 is abundant, highly antigenic, and it is recognized by the sera of most NCC patients. In addition, the antibody binds to other highly antigenic GPs including GP16, GP18, GP24, GP28 and GP34. To determine the type of cells taking up the parasite GCs, human infected tissue sections were simultaneously stained with anti-GP12 and antibodies against immune cells (macrophages, CD3 T cells, CD8 T cells, B cells, and mast cells). Two types of human infected lesions were analyzed, the first were characterized by a mild inflammatory response without granuloma formation. In these tissues anti-GP12 labels the whole tegument (Figure 7A), and it appears to be released as macrophages surrounding the metacestode showed positive staining (Figure 7A). In contrast, anti-GP12 was not found in CD8 T cells (Figure 7B), B cells (Figure 7C) and mast cells (not shown). Interestingly, GP12 was consistently detected in the tegument of the metacestodes analyzed (Figure 7A). The second type of lesion showed a strong inflammatory response accompanied of a granulomatous reaction [11] and macrophage-like cells were also the only cell type tested that was positive for GP12 (Figure 7D). In addition, macrophages referred to as epithelioid histiocytes are organized as a layer around the metacestode and in certain areas have fused forming giant cells. Macrophages and giant cells have a great number of vacuoles that vary in size and that display positive staining for anti-GP12 (Figure 7E). Anti-GP12 staining was also detected in parasite remnants detected in these lesions. In addition, macrophages and giant cells (Figure 7F) around the metacestode displayed low expression of MHC-II molecules (Figure 7G). The expression of MHC-II appears to be higher in cells located further away from the parasite (Figure 7G) suggesting that molecules released by the parasite may downregulate MHC-II.

## Discussion

In this study we describe the composition, changes and release during infection of the *T. solium* and *M. corti* tegument. The results show that the tegument of these helminths changes morphologically and biochemically as the parasites interact with the host microenvironment. Early in the infection, IB4 bound molecules are lost from the tegument whereas molecules bound by WGA are continuously released from this structure during infection. In addition, the material released from the tegument is found in infiltrating leukocytes that we have characterized extensively in previous studies [6], [7], [9]–[11]. The mechanisms used by *T. solium* metacestodes to establish a chronic infection in the human CNS remain poorly elucidated. Because of their macroscopic size, helminths utilize elaborate mechanisms to manipulate the host immune response and to ensure long term survival. One of the structures involved in sustaining such immunoregulation appears to be the tegument that is subject to damage under host response [32]. Analyses of *M. corti* metacestodes injected in the mouse brain revealed that the tegument of these parasites is partially lost as the parasite moves into the CNS. This is consistent with studies in trematodes like *Schistosoma sp.,* in which the first larval form or miracidium is covered with a thick glycocalix coat that after penetration in the intermediate host gets considerably thinner [33].

CNS infection with *M. corti* metacestode labeled with hydrazide-Alexa 488 showed that after 12 and 24 hours, the molecules present in the distal cytoplasm and proximal cytoplasm of these parasites are released and detected in host cells located in internal and external leptomeninges. In addition, the degree of penetration or contact of *M. corti* metacestode appears to be directly correlated with the extent of material released from the tegument. Lectin staining confirmed the data obtained with the hydrazide-Alexa 488 and allowed us to determine that a large proportion of the molecules lost from the tegument early in the infection are rich in acetyl-D-galactosamine ends and α-D-galactosyl residues as shown by IB4 binding. Interestingly, the GCs bound by this lectin disappear from areas of parasite-host contact and are essentially undetected on the parasite's tegument after 1 wk and throughout later post-infection times. Analyses of the lectin binding molecules in *T. solium* tegument showed a very similar pattern of release in porcine and human infected samples validating the data obtained in the mouse model. The GCs labeled with hydrazide-alexa 488 and recognized by IB4 appear to be involved in the first response generated by these organisms in the CNS microenviroment as they are phagocytosed by immune cells. However the IB4 binding molecules are no longer associated with the parasite. These results correlate with the morphological changes detected in the tegument of metacestodes penetrating the CNS as early as 24 hours PI. Most of the parasites displayed a reduction in the number of discoidal bodies, microtriches and mitochondria along with the appearance of residual and autophagic-like bodies, perhaps indicative of nutrient acquisition by recycling cytosolic material. The rapid release of tegument material upon host penetration correlated with the loss of IB4 bound GCs and associates with the reduced number of discoidal bodies and mitochondria. These changes suggest a temporary reduction in the rate of tegument build up and thus, less antigenic exposure in the initial steps of parasite invasion, particularly antigens that bind IB4. It has been proposed in the case of *S. mansoni* that organelle alterations and release of tegument material are part of the counterattack to the host response as surface bound antibodies, complement and activated immune effector cells can be sloughed off [34].

IB4 and GCs bound to hydrazide-Alexa 488 appear to play an important role in the evasion of the early stages of host response, but NCC and many other parasitic diseases are long-lasting, chronic infections in which the microorganisms persist over long periods [2],[35]. Therefore, additional mechanisms to evade or modulate continuing host immune responses are likely involved in NCC. In contrast to the early disappearance of IB4 bound GCs, WGA bound GCs present in the tegument before infection were detected during several previously characterized stages of murine, porcine and human NCC. Samples infected with *T. solium* also showed continuous release of ConA bound GCs at various times post infection. The amount of these antigens likely affects the outcome of the host response as molecules highly glycosylated are known to interfere in a concentration dependant manner with the antigen presentation process in cells like macrophages and dendritic cells [36]. In the NCC samples analyzed, parasite-derived GCs were detected in numerous cells surrounding the parasites. We have shown previously that infiltrating leukocytes include macrophages, dendritic cells, B cells and γδ T cells [7], [9]–[11]. Inhibition of the immune response correlates with the apparent lack of damage to many of the metacestodes surrounded by phagocytic cells. This is supported by the low to undetectable levels of MHC-II found in epithelioid histiocytes surrounding *T. solium* metacestodes lodged in human CNS. Likewise, dendritic cells stimulated with helminth components show limited upregulation of CD80, CD86 and MHC-II expression in comparison to the expression induced by bacterial antigens [37]. In some studies has been shown that NCC patients exhibit a depressed peripheral cellular immune response [38], although this is controversial [39],[40]. In other helminth infections, results indicate that released products may interfere with the generation of pro-inflammatory mediators [41], the antigen presentation process [42] and other immune defense mechanisms elicited by the host upon helminth infection [3],[43],[44].

The continuous release of parasite GCs and the establishment of equilibrium between host response and parasite infection also implies a homeostatic state that would require robust immunoregulatory mechanisms. This may be particularly important as we have shown previously that *M. corti* actively secretes proteins [45] although the potential carbohydrate moieties of secreted molecules were not explored. Thus, both active and passive mechanisms of antigen release may have to be controlled to maintain the host-parasite balance. Consistent with this, the granulomatous response in human NCC has a strong immunoregulatory component involving the expression of IL-10 and TGF-β [11]. In the host response against helminth infections, the expression of such immunoregulatory cytokines has been associated with a gradual exhaustion of the immune response in terms of T-cell proliferation and production of inflammatory cytokines [42]. Interestingly, peripheral blood mononuclear cells stimulated with *Taenia sp.* GCs elicit IL-10, TGF-β and molecules associated with anti-inflammatory responses [46] and this type of immune reaction is known to be inversely associated with the severity of infection in human NCC [38]. The hyporesponsive status developed in helminth infections as NCC can be modified when the immunoregulatory state is suppressed. In a murine model of schistosomiasis the reduction of immunoregulatory cells resulted in increased killing of parasites and enhancement of Ag-specific immune responses [47]. Thus, the constant release and persistence of parasite GCs during the course of NCC most likely leads to a suppressive and immunoregulatory environment that supports parasite establishment and maintenance while minimizing damaging inflammatory responses. Death of the parasite may eliminate this balance and would be consistent with known adverse inflammatory reactions when patients are treated with anti-helminth drugs.

In parallel, a portion of GCs released during the course of NCC may enter the CSF circulation and divert immune cells to static deposits of antigens located in leptomeninges, ventricles and areas far from the parasite. These areas are characterized by the presence of inflammatory cells, proinflammatory mediators and moderate expression of MHC-II and costimulatory molecules [5],[7],[11] which contrast with the diminished display of antigen presentation associated molecules in cells proximal to the parasite. Such inflammatory responses may be responsible for the neurological symptoms seen in patients as well as infected mice harboring viable organisms.

Our current research is to isolate and characterize the major GCs with distinct release patterns described herein. We anticipate that some GCs will be immunostimulatory and others immunosuppressive and that the balance of such molecules will dictate the course and severity of NCC.
